# A study of the correlations between IVIM-DWI parameters and the histologic differentiation of hepatocellular carcinoma

**DOI:** 10.1038/s41598-021-89784-2

**Published:** 2021-05-17

**Authors:** Yi Zhou, Gang Yang, Xue-Qin Gong, Yun-Yun Tao, Ran Wang, Jing Zheng, Cui Yang, Juan Peng, Lin Yang, Jing-Dong Li, Xiao-Ming Zhang

**Affiliations:** 1 Medical Imaging Key Laboratory of Sichuan Province, Medical Research Center, Department of Radiology, Affiliated Hospital of North Sichuan Medical College, Nanchong, 637000 Sichuan People’s Republic of China; 2 Institute of Hepato-Biliary-Intestinal Disease, Department of Hepatobiliary Surgery, Affiliated Hospital of North Sichuan Medical College, Nanchong, 637000 Sichuan People’s Republic of China; 3 Department of Radiology, People’s Hospital of Deyang City, Deyang, 618000 Sichuan People’s Republic of China

**Keywords:** Hepatocellular carcinoma, Diagnostic markers

## Abstract

The present study aimed to investigate the value of intravoxel incoherent motion diffusion weighted imaging (IVIM-DWI) in the preoperative prediction of the histologic differentiation of hepatocellular carcinoma (HCC). Seventy HCC patients were scanned with a 3.0 T magnetic resonance scanner. The values of apparent diffusion coefficient (ADC), slow apparent diffusion coefficient (D), fast apparent diffusion coefficient (D*), and the fraction of the fast apparent diffusion coefficient (f) were measured. Analysis of variance was used to compare the differences in parameters between groups with different degrees of histologic differentiation. *p* < 0.05 was considered statistically significant. Receiver operating characteristic (ROC) curves were used to analyse the efficacy of IVIM-DWI parameters for predicting the histologic differentiation of HCC. The ADC and D values for well, moderately and poorly differentiated HCC were 1.35 ± 0.17 × 10^−3^ mm^2^/s, 1.16 ± 0.17 × 10^−3^ mm^2^/s, 0.98 ± 0.21 × 10^−3^ mm^2^/s, and 1.06 ± 0.15 × 10^−3^ mm^2^/s, 0.88 ± 0.16 × 10^−3^ mm^2^/s, 0.76 ± 0.18 × 10^−3^ mm^2^/s, respectively, and all differences were significant. The D* and f values of the three groups were 32.87 ± 14.70 × 10^−3^ mm^2^/s, 41.68 ± 17.90 × 10^−3^ mm^2^/s, 34.54 ± 18.60 × 10^−3^ mm^2^/s and 0.22 ± 0.07, 0.23 ± 0.08, 0.18 ± 0.07, respectively, with no significant difference. When the cut-off values of ADC and D were 1.25 × 10^−3^ mm^2^/s and 0.97 × 10^−3^ mm^2^/s, respectively, their diagnostic sensitivities and specificities for distinguishing well differentiated HCC from moderately differentiated and poorly differentiated HCC were 73.3%, 85.5%, 86.7%, and 78.2%, and their areas under the ROC curve were 0.821 and 0.841, respectively. ADC and D values can be used preoperatively to predict the degree of histologic differentiation in HCC, and the D value has better diagnostic value.

## Introduction

Hepatocellular carcinoma (HCC) is a malignant tumour with the sixth highest morbidity rate and the fourth highest mortality rate worldwide and causes a high disease burden^1,2^. Surgical treatment is the most important means of increasing the long-term survival of HCC patients^3^. The histologic differentiation of HCC is an important factor affecting the prognosis of patients^4–8^. Preoperative prediction of the histologic differentiation of HCC using imaging methods is very important for the development of therapeutic strategies.

The traditional diffusion-weighted imaging (DWI) parameter, namely, the apparent diffusion coefficient (ADC), can quantitatively reflect the diffusion motion of water molecules in active tissues^9^. However, ADC values include two types of information: the molecular diffusion of water and microcirculation perfusion^10^. Intravoxel incoherent motion (IVIM) has emerged in recent years and can distinguish between the diffusion of pure water molecules and microcirculation perfusion-related information in tissues, thus compensating for the deficiency of traditional DWI^11,12^. Currently, IVIM-DWI studies of hepatic lesions mainly focus on the staging of hepatic fibrosis^13–16^, identification of hepatic nodules^17–22^, and evaluation of the treatment response of liver cancer^23–27^. However, studies of IVIM-DWI and histologic differentiation in HCC are rare, and the results of the few available studies are inconsistent.

Therefore, this study analysed correlations between IVIM-DWI parameters and histologic differentiation in 70 HCC patients who underwent surgical resection at our hospital between September 2017 and September 2019. We aimed to investigate the value of IVIM-DWI for preoperative prediction of the histologic differentiation of HCC.

## Materials and methods

### Case collection

The present study was approved by the Ethics Committee of the Affiliated Hospital of North Sichuan Medical College. Written informed consent was obtained from all individual participants, and all methods were performed in accordance with the relevant guidelines and regulations. The records of the 70 patients who underwent surgical resection of HCC between September 2017 and September 2019 at our hospital were collected. The patients included 65 males and 5 females aged from 24 to 70 years, with a mean age of 50.64 ± 10.51 years. The findings were validated in an independent cohort (22 patients, age: 53.31 ± 12.49 years).

### Magnetic resonance imaging (MRI) scans

All patients were scanned with the same GE Discovery MR750 3.0 T magnetic resonance scanner (GE Medical Systems, Milwaukee, Wis., USA). Body-specific 32-channel phased-array coils were used. Plain liver MRI, IVIM-DWI, and dynamic enhancement scans were performed. The scanning sequences and parameters were the same as those used in our previous studies^22^.

### Image postprocessing

MRI results were sent to a GE ADW4.6 postprocessing workstation, and Function-MADC software was used for image analysis. The region of interest (ROI) was placed in the largest solid area of the tumour showing the main properties of the tumour and was compatible with the pathological evaluation area while avoiding haemorrhagic and necrotic areas. Pseudocolour maps of the ADC, slow apparent diffusion coefficient (D), fast apparent diffusion coefficient (D*), and the fraction of the fast apparent diffusion coefficient (f) were generated, and the ADC, D, D*, and f values were measured. The values of all parameters were measured three times, and the average value was recorded.

### Histologic grading

All surgically resected tumours were fixed with formalin, embedded in paraffin, and stained with haematoxylin–eosin (HE). Histologic differentiation was classified according to the criteria of the World Health Organization (WHO) Classification of Tumors: Well differentiated: tumour cells resembling mature hepatocytes with minimal to mild atypia, cytoplasm ranging from abundant and eosinophilic to moderate and basophilic, and nuclei showing minimal to mild nuclear atypia; moderately differentiated: clearly malignant cells according to HE staining with a morphology strongly suggesting hepatocellular differentiation, cytoplasm ranging from abundant and eosinophilic to moderate and basophilic, and nuclei showing moderate nuclear atypia; and poorly differentiated: clearly malignant cells on HE staining with a morphology consistent with the broad spectrum of poorly differentiated carcinomas, cytoplasm ranging from moderate to scarce, which is usually basophilic, and nuclei with marked nuclear pleomorphism, which may include anaplastic giant cells. The number of slides for pathological study was determined according to the tumour volume (one slide per centimetre). When heterogeneous areas exist in one tumour nodule or between different nodules, the tumour grade is defined according to the major grade of tumour tissue.

### Statistical analysis

Analysis of variance (ANOVA) was used to compare differences in parameters among groups with different degrees of histologic differentiation. The Spearman rank correlation test was used to analyse the correlations between the IVIM-DWI parameters and the histologic differentiation of HCC tissues*.* A value of *p* < 0.05 was considered statistically significant. Receiver operating characteristic (ROC) curves were used to analyse the efficacy of IVIM-DWI parameters for predicting the histologic differentiation of HCC.

## Results

Plain MRI scans of HCC showed slightly longer T1-weighted imaging (T1WI) and T2WI values with heterogeneous signals, and some lesions showed long T1 and T2 values for necrotic liquefaction areas. Most of the lesions showed "fast-in fast-out" intensification on the enhanced scan, while a few lesions showed mild, uneven intensification or no obvious intensification in the arterial phase.

**Figure 1:**
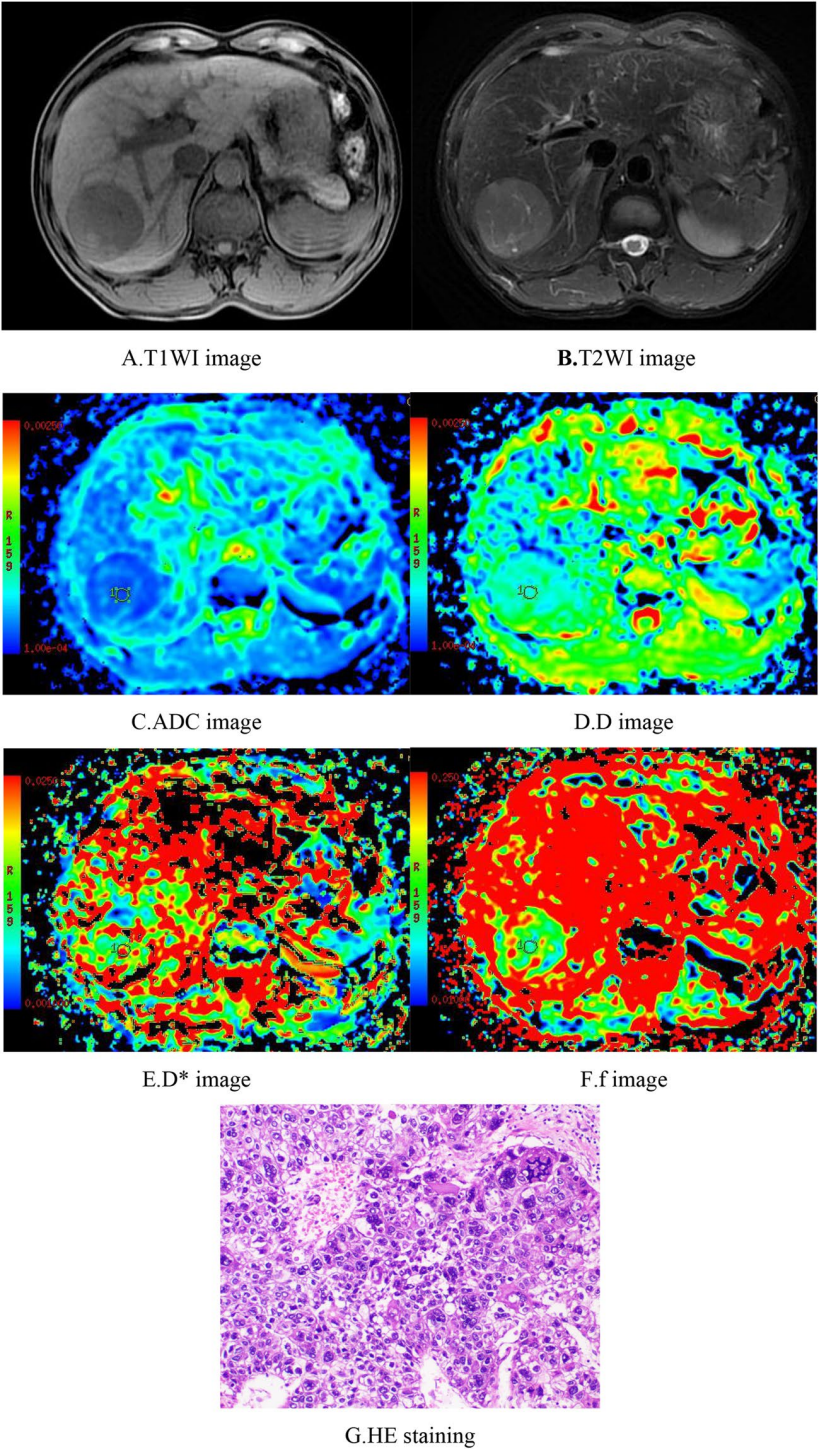
Female, 50 years old, HCC in the right lobe, well differentiated. (**A**) T1WI image. Low-signal area is visible in the right hepatic lobe with a clear boundary. (**B**) T2WI images. The lesion showed a heterogeneous, slightly high signal and a clear boundary. (**C**–**F**) ADC, D, D*, and f images; the measured values were 1.56 × 10^−3^ mm^2^/s, 1.04 × 10^−3^ mm^2^/s, 15.50 × 10^−3^ mm^2^/s, and 0.29, respectively. (**G**) HE staining (*100) showed well differentiated HCC tissues.

**Figure 2:**
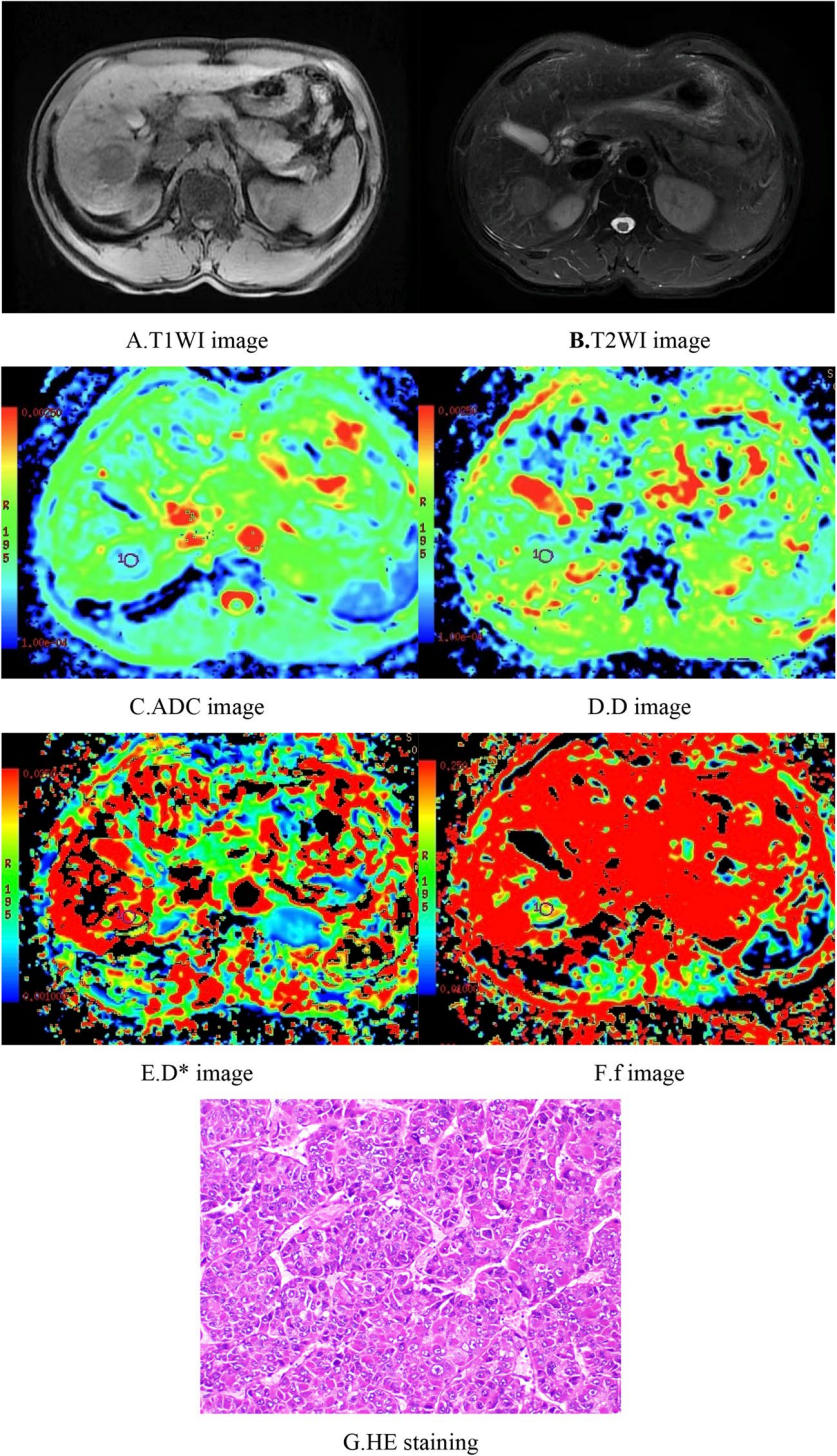
Male, 61 years old, HCC in the right hepatic lobe. (**A**) T1WI image. The lesion showed a low signal and a relatively clear boundary. (**B**) T2WI image.The lesion showed a slightly high signal, with a relatively clear boundary. (**C**–**F**) ADC, D, D*, and f images, respectively. The ADC value was 1.18 × 10^−3^ mm^2^/s, the D value was 0.92 × 10^−3^ mm^2^/s, the D* value was 30.8 × 10^−3^ mm^2^/s, and the f value was 0.18. G. HE staining (*100) showed moderately differentiated HCC tissues.

**Figure 3:**
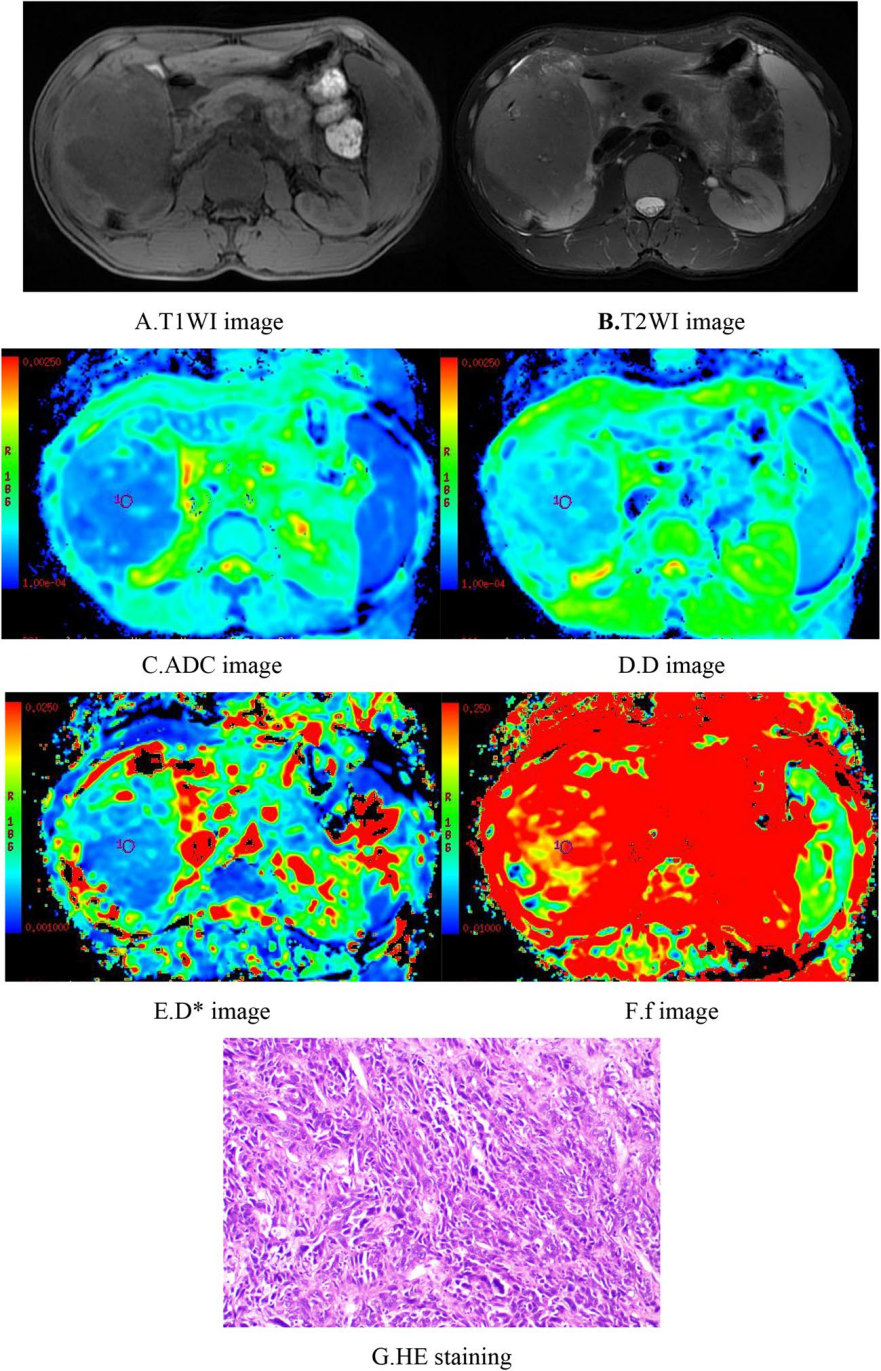
Male, 29 years old, HCC in the right hepatic lobe. (**A**) T1WI image. The lesion showed a heterogeneous low signal, an irregular morphology, and an unclear boundary. (**B**) T2WI image. The lesion showed a heterogeneous, slightly high signal, an unclear boundary, and patchy necrosis inside. (**C**–**F**) ADC, D, D*, and f images, respectively. The measured ADC was 1.10 × 10^−3^ mm^2^/s, the D value was 0.86 × 10^−3^ mm^2^/s, the D* value was 29.90 × 10^−3^ mm^2^/s, and the f value was 0.16. (**G**) HE staining (*100) showed poorly differentiated HCC tissues.

**Figure 4:**
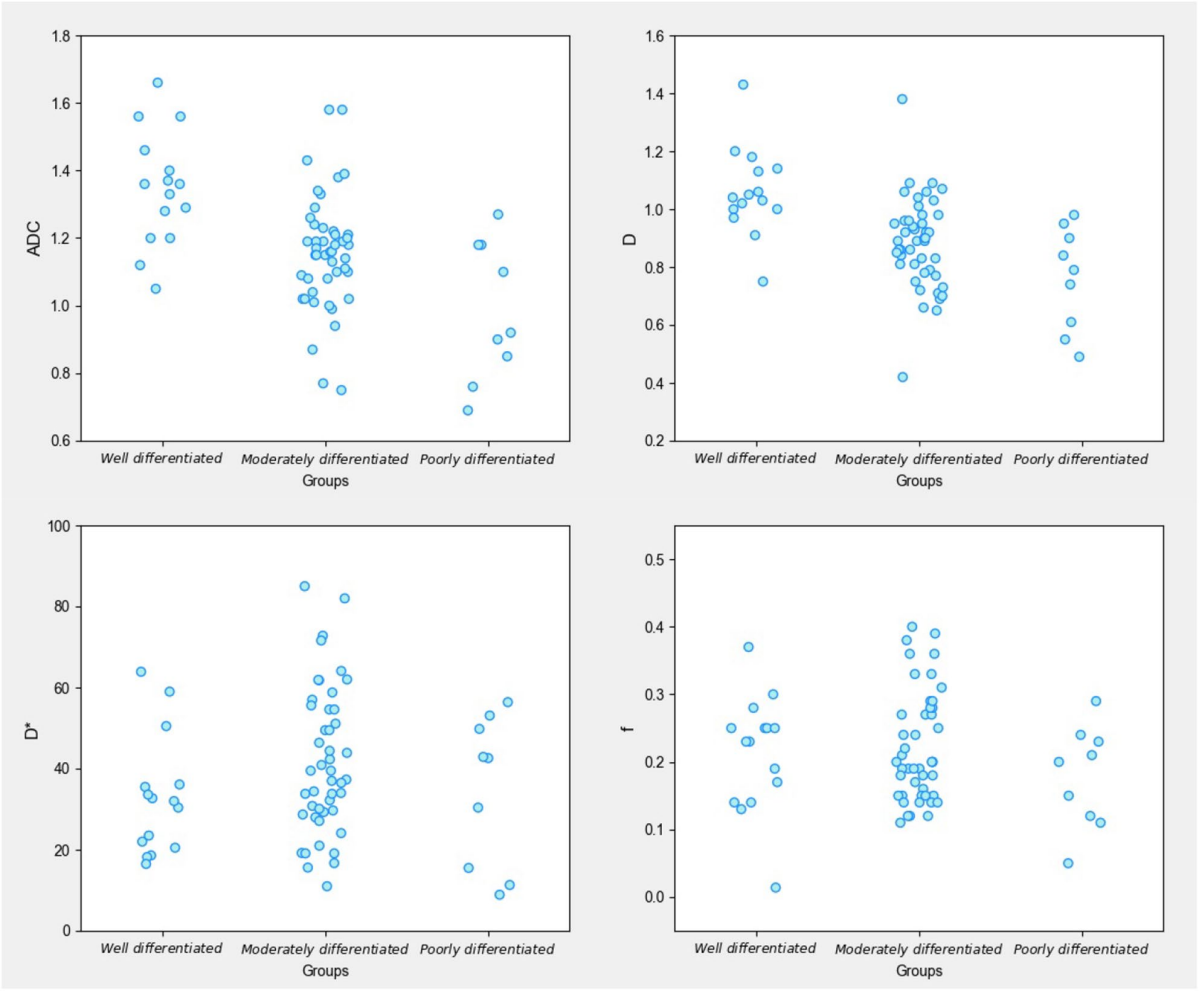
Scatter plots showing IVIM-DWI parameters in different HCC histologic differentiation groups.The graphs were created by using Python 3.6 (https://www.python.org/).

**Figure 5:**
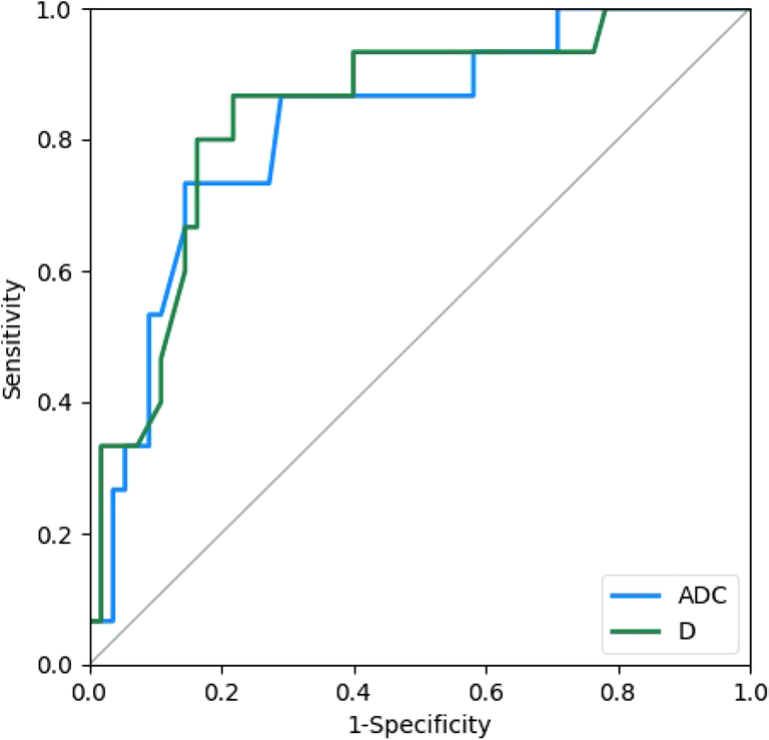
The efficacy of IVIM-DWI parameters for the diagnosis of well differentiated HCC. The ROC curves were created by using Python 3.6 (https://www.python.org/).

Among the 70 cases of HCC, 15 were well differentiated, 46 were moderately differentiated, and 9 were poorly differentiated. The ADC values for well, moderately, and poorly differentiated HCC were 1.35 ± 0.17 × 10^−3^ mm^2^/s, 1.16 ± 0.17 × 10^−3^ mm^2^/s, and 0.98 ± 0.21 × 10^−3^ mm^2^/s, and the D values were 1.06 ± 0.15 × 10^−3^ mm^2^/s, 0.88 ± 0.16 × 10^−3^ mm^2^/s, and 0.76 ± 0.18 × 10^−3^ mm^2^/s, respectively; all differences were significant. The well differentiated group had significantly higher ADC and D values than the moderately differentiated and poorly differentiated groups, and the moderately differentiated group had significantly higher ADC and D values than the poorly differentiated group. ADC and D values were significantly associated with histologic differentiation (r =  − 0.510, *p* < 0.05 and r =  − 0.512, *p* < 0.05, respectively). The D* values of the well, moderately, and poorly differentiated HCC groups were 32.87 ± 14.70 × 10^−3^ mm^2^/s, 41.68 ± 17.90 × 10^−3^ mm^2^/s, and 34.54 ± 18.60 × 10^−3^ mm^2^/s, and the f values were 0.22 ± 0.07, 0.23 ± 0.08, and 0.18 ± 0.07, respectively, with no significant differences; the D* and f values were not significantly correlated with histologic differentiation. When the cutoff values of the ADC and D were 1.25 × 10^−3^ mm^2^/s and 0.97 × 10^−3^ mm^2^/s, respectively, their diagnostic sensitivities and specificities for distinguishing well differentiated HCC from moderately differentiated and poorly differentiated HCC were 73.3% and 85.5% (ADC) and 86.7% and 78.2% (D), and their areas under the ROC curves (AUCs) were 0.821 and 0.841, respectively. The AUC was larger for D (Tables 1, 2) (Figs. 1, 2, 3, 4, 5).

**Table 1:** Comparison of IVIM-DWI parameters in different HCC histologic differentiation groups.

Parameters	Well differentiated group	Moderately differentiated group	Poorly differentiated group	F	*p*
ADC	1.35 ± 0.17^a^	1.16 ± 0.17^b^	0.98 ± 0.21^c^	13.156	0.000
D	1.06 ± 0.15^a^	0.88 ± 0.16^b^	0.76 ± 0.18^c^	11.586	0.000
D*	32.87 ± 14.70	41.68 ± 17.90	34.54 ± 18.60	1.777	0.177
f	0.22 ± 0.07	0.23 ± 0.08	0.18 ± 0.07	1.207	0.306

The units for ADC, D, and D* were all × 10^−3^ mm^2^/s. Different superscript letters indicate significant differences in the parameters between various groups.

**Table 2:** The efficacy of IVIM parameters for distinguishing well differentiated HCC from moderately and poorly differentiated HCC.

Parameters	Cutoff	Sensitivity (%)	Specificity (%)	AUC	95% CI
ADC	1.25	73.3	85.5	0.821	0.702–0.939
D	0.97	86.7	78.2	0.841	0.727–0.954

*CI* confidence interval.

In the independent validation cohort, the ADC and D values of the three groups were also significantly different and showed the value of distinguishing well differentiated HCC from moderately differentiated and poorly differentiated HCC; the D value was high and had an AUC of 0.896. The results are consistent with those of the previous cohort.

## Discussion

Studies have shown that the ADC values of traditional DWI and the histologic grade are significantly negatively correlated in HCC^5,28,29^. However, reports show inconsistent conclusions. Nasu et al.^10^ showed that the mean ADC values of well differentiated, moderately differentiated, and poorly differentiated HCC were 1.45, 1.46, and 1.36 × 10^−3^ mm^2^/s, respectively. The ADC had no significant correlation with the histologic grade; however, as the pathological grade of HCC increased, the DWI signal also increased.

Few studies are available on the correlations between IVIM-DWI parameters and histologic differentiation in HCC; in the few available studies, the results are inconsistent. Some studies have shown that ADC and D values have significant negative correlations with the histologic grade in HCC and that ADC and D values can be used for histologic grading, while D* and f values did not significantly correlate with the histologic grade. For example, Woo et al.^5^ investigated the IVIM-DWI results of 40 HCC patients and found that the D value and ADC value of HCC in the high-grade group were significantly lower than those in the low-grade group; ROC analysis demonstrated a higher AUC value for D than for ADC for differentiating high-grade HCC from low-grade HCC. Wei et al.^30^ also reported similar results.

Other studies showed that in addition to ADC and D values, D* and/or f values were also associated with the histologic grade. Zhu et al.^31^ studied the IVIM-DWI results in 62 cases of HCC and found significant differences in the ADC and D values among the three groups (G1, G2, and G3), and ADC, D, and D* values were significantly correlated with the histologic grade. Shan^32^ used ADC and IVIM-DWI parameters to evaluate histologic differentiation in 106 cases of hepatitis B virus-related HCC. The results showed that the ADC, D, and f values of HCC were significantly different among the well differentiated, moderately differentiated, and poorly differentiated groups and were significantly correlated with histologic differentiation; the AUCs for the diagnosis of well differentiated HCC by the ADC, D, and f were 0.903, 0.84, and 0.782, respectively, while the AUCs for the diagnosis of poorly differentiated HCC were 0.787, 0.726, and 0.624, respectively. Sokmen^33^ and Granata et al.^34^ obtained similar results. In addition, Li^35^ studied IVIM-DWI in rat models of HCC induced by diethylnitrosamine (DEN). The results showed that, compared with the low histologic grade group, the ADC and D in the high-grade group were significantly decreased, while D* and f were significantly increased. This study suggested that the ADC and IVIM parameters had equal value for predicting the histologic grade of HCC. The ADC and all IVIM parameters can be used for the pathological grading and preoperative assessment of HCC.

In this study, the ADC values of HCC tissues were all higher than the D values, which is similar to previous literature reports^5,17,18^. The reason may be that the ADC value obtained using the single exponential model not only includes information about water molecule diffusion but also contains information related to microcirculation perfusion. In the present study, significant differences in the ADC and D values were found among different HCC histologic differentiation groups; histologic differentiation was significantly correlated with the ADC and D values, and poorer histologic differentiation resulted in lower ADC and D values. The possible reason for these results may be that poorer tumour differentiation led to faster proliferation, resulting in increases in the number of tumour cells and the tumour density and a decrease in the amount of intercellular substance; therefore, the diffusion of water molecules within the tumour tissue is more constrained, which is reflected by significant decreases in ADC and D values^11^. IVIM-DWI distinguishes between simple water molecule diffusion information and microcirculatory perfusion diffusion information within the tissues, thereby better reflecting the microstructural changes within the tissue^36^. The results of this study showed that the D value was more effective for identifying the histologic differentiation of HCC.

Both the D* and f values can reflect microcirculatory perfusion-related information in tissues. The D* value reflects the diffusion motion of microcirculation perfusion in the tissue capillary network, which is related to the morphology of capillaries and the flow velocity of blood inside the capillaries. The f value represents the volume ratio of microcirculation perfusion-related diffusion to total diffusion. It is affected by the capillary vascular capacity within the tissues^37^. The D* and f values in this study were not significantly correlated with histologic differentiation, which is consistent with the results of Woo et al.^5^, whereas some differences from the results of Li^35^ and Granata et al.^34^ were noted.

The inconsistency of the above results may be related to the following factors: (1) different case compositions: Yoon et al.^18^ showed that the D* and f values of hepatic lesions with a rich blood supply were higher than those of lesions with a poor blood supply. Woo et al.^5^ found that the f value was positively correlated with the enhancement ratio in the arterial phase of the tumours. In this study, some of the HCC lesions were characterized by a lack of blood supply, with no obvious enhancement or only mild heterogeneous enhancement in the hepatic arterial phase, which may offset the high D* and f value effects of lesions with a rich blood supply. (2) Tumour heterogeneity: A higher tumour histologic grade results in greater variability and a more complicated microstructure, leading to greater differences in the D* and f values. (3) The microcirculation perfusion represented by the D* value might also include other physiological processes, such as physiological activities including glandular secretion, as well as fluid flow inside the glandular ducts and catheters^38^; the activities of these fluids are difficult to differentiate from blood perfusion, which may affect the measurement of the D* and f values. (4) The D* and f values have poor repeatability^39^.

The limitations of this study are as follows: (1) the sample sizes were quite different among groups. In particular, the small number of cases in the poorly differentiated group may have led to bias in the results. The sample size should be increased in the future to continue the study. (2) Manual delineation of the ROIs led to certain unavoidable measurement errors. (3) A consensus regarding the number and distribution of b values is still lacking. Different b values and distributions may result in differences in parameter measurements^12,13,40,41^.

In summary, the ADC and D values of HCC tissues were significantly correlated with histologic differentiation. ADC and D values can be used to preoperatively predict the degree of histologic differentiation of HCC, and the D value has better diagnostic value.
